# Neutrophil to lymphocyte ratio and C-reactive protein level as prognostic markers in mild versus severe COVID-19 patients 

**Published:** 2020

**Authors:** Seyed Dawood Mousavi-Nasab, Rajab Mardani, Hosein Nasr Azadani, Fatemeh zali, Abbas Ahmadi Vasmehjani, Shahram Sabeti, Ilad Alavi Darazam, Nayebali Ahmadi

**Affiliations:** 1 *Viral vaccine research center, Pasteur Institute of Iran, Tehran, Iran *; 2 *Department of Research and Development, Production and Research Complex, Pasteur Institute, Tehran, Iran*; 3 *Department of Biochemistry, Pasteur Institute of Iran, Tehran, Iran*; 4 *Department of Virology, School of Public Health, Tehran University of Medical Sciences, Tehran, Iran*; 5 *Department of Clinical Biochemistry, Faculty of Medicine, Tehran University of Medical Science, Tehran, Iran*; 6 *Pathology Ward, Loghman Hakim Hospital, Shahid Beheshti University of Medical Sciences, Tehran, Iran*; 7 *Infectious Diseases and Tropical Research Center, Loghman Hakim Hospital, Shahid Beheshti University of Medical Sciences, Tehran, Iran*; 8 *Proteomics Research Center, Department of Medical Lab Technology, Faculty of Paramedical Sciences, Shahid Beheshti University of Medical Sciences, Tehran, Iran*

**Keywords:** SARS-CoV-2, neutrophil‐to‐lymphocyte ratio (NLR), CRP, COVID-19

## Abstract

**Aim:**

This research aimed to investigate neutrophil‐to‐lymphocyte ratio (NLR) with C-reactive protein to identify potential clinical predictors and analyze differences among severe and non-severe COVID-19 patients.

**Background:**

NLR and CRP are established markers that reflect systemic inflammatory, and these parameters alter in patients with novel coronavirus (SARS-CoV-2) pneumonia (COVID-19).

**Methods:**

A population of patients with COVID-19 referred to Loghman Hospital in Tehran was analyzed. The baseline data of laboratory examinations, including NLR and CRP levels, was collected. Pearson analysis was used to assess the independent relationship between the NLR with disease severity and CRP levels.

**Results:**

COVID-19 cases comprised 14 (20%) patients with severe disease and 56 (80%) with non-severe infection. The mean values of WBC, NEU, LYM, and NLR of the severe patients were significantly higher than those of the non-severe patients. Forty-six patients (65.7%) had NLR >1, and the remaining patients had NLR <1. Plasma CRP levels were higher in severe cases than in non-severe cases, and this difference was significant. The results showed that NLR was positively correlated with CRP levels (R=0.23) and negatively correlated with WBC (R=-0.38). CRP (AUC = 0.97, 95% CI: 0.95-0.99) and NLR (AUC = 0.87, 95% CI: 0.81-0.93) had very good accuracy in predicting the severity of COVID-19 disease.

**Conclusion:**

The findings of this study indicated that the integration of NLR and CRP may lead to improved predictions and is recommended as a valuable early marker to assess prognosis and evaluate the severity of clinical symptoms in COVID-19 patients.

## Introduction

 In December 2019, a novel betacoronavirus outbreak was identified in Wuhan, China (1). Compared with other betacoronaviruses, it was characterized as a highly contagious and deadly strain (2). The International Committee on Taxonomy of Viruses (ICTV) named the virus causing the current outbreak Severe Acute Respiratory Syndrome Coronavirus-2 (SARS-CoV-2) (3). SARS-CoV-2 is mainly transmitted between people by aerosol and contact routes (4). COVID-19 is a disease characterized by diffuse inflammation changes in the lungs (5). However, its pathogenesis, clinical features, and pathological changes are still being explored, especially in severely ill patients with complicated conditions, multi-organ complications, long disease course, and high mortality (6). Commonly, severe patients are moved to the ICU for treatment, while mild patients can isolate themselves either at home or in larger community facilities (7). Predicting severe patients at an early stage is of great clinical significance in reducing clinical morbidity and enhancing the treatment process for COVID-19 pneumonia (8). Hence, early differential diagnosis and prediction of the severity of SARS‐COV‐2 infection is needed. C-reactive protein (CRP) is a widely used diagnostic marker primarily used to assess ongoing inflammation. It appears in blood within 6–10 hours of any tissue damaging event and decreases exponentially over 18–20 h (9, 10). The complete blood count is the most available, efficient, and economic examination. Moreover, peripheral white blood cell (WBC) count and neutrophil (NEU)-to-lymphocyte (LYM) ratio (NLR) are indicators of the systematic inflammatory response (11, 12). Several previous studies have been performed on neutrophils, lymphocytes, and CRP in COVID‐19 patients (13, 14), but little is known about their association with disease severity in Iran. More studies are needed to validate the findings of previous reports in different populations. This study aimed to investigate NLR with CRP to identify potential clinical predictors and analyze differences among severe and non-severe COVID-19 patients. 

## Methods

**Participants**

This single institution, retrospective study was conducted on recruited patients who referred to Loghman Hospital, Tehran, Iran, from March 26 to April 21, 2020. Approval was obtained from the ethics board of Shahid Beheshti University of Medical Sciences. Positive COVID-19 patients were classified into two groups based on their clinical symptom results (non-severe and severe). According to clinical reports, 56 patients were listed as moderate and 14 had developed into severe infection (***intensive care unit*** (***ICU***) admission). Data for each patient, such as age and gender, was collected from medical records. Blood samples were collected for laboratory assessments, including CBC with differential count and CRP levels.

**Data gathering and laboratory data**

Demographic data, including age, gender, and clinical data, was recorded. Blood samples were collected from each participant and routine blood tests including white blood cell (WBC) count, lymphocyte (LYM) count, and neutrophil (NEU) count were performed. Inflammation biochemistry parameters such as CRP were also assessed using a CRP immunoturbidometric PARS Azmon kit on a HITACHI 7600-020 automated biochemistry analyzer.

**Statistical analysis**

Statistical analysis was performed using GraphPad Prism, version 8.0 (GraphPad Software, In, San Diego, USA). Measurement data with normal distribution is represented as mean ± standard deviation (mean ± SD). For continuous variables that were normally distributed, differences between two groups were compared using the χ^2^ and t tests. Correlation analysis was calculated by Spearman. A *p-*value < 0.05 was considered to be statistically significant. In this study, AUC 0.9 to 1 was defined as excellent accuracy, 0.8 to 0.9 as very good, 0.7 to 0.8 as good, 0.6 to 0.7 as sufficient, 0.5 to 0.6 as bad, and < 0.5 as poor (useless test). 

## Results

**Characteristics of the admitted cases in the study subjects**

COVID-19 cases with the mean age of 42.7 ± 12.4 (range: 19-78) years were studied. Among them, 40.2% were in the age range of 30 to 49 years. Forty (57.1%) cases were male and the remaining were female. Fourteen (20%) patients had severe disease, and 56 (80%) had non-severe COVID-19.

**Laboratory parameters**

**Figure 1:**
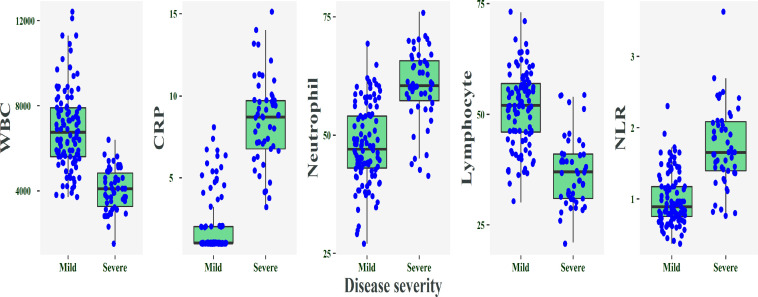
The distribution of WBC, NEU, LYM, CRP, and NLR based on disease severity. The data showed that 10 (71.4%) severe and 4 (8.9%) non-severe cases had neutrophilia and 12 (85.7%) severe and 6 (10.7%) non-severe cases had lymphopenia. The laboratory reference values of LYM and NEU were 1.2–3.4 and 1.8–6.3 E9/L, respectively

Table 1 and Figure 1 show the distributions of WBC, NEU, LYM, CRP and NLR between the two COVID-19 patient groups. There were no significant differences between two groups in gender and age.

**Table 1:** Laboratory parameter findings of severe and non-severe COVID-19 patients

Variables ^a^	All patients (n=70)	Severe (n=14)	Non-severe (n=56)	*P*
Sex: ^b^				
Male Female	40 (57.1%) 30 (42.9%)	11 (78.5%) 3 (21.5%)	29 (51.8%) 27 (48.2%)	0.07
Age	42.7 ± 12.4	41.3 ± 16.5	43.0 ± 11.4	0.65
WBC (cells/mm^3^)(M ± SD)	6.93 ± 3.9	9.1 ± 5.6	6.4 ± 2.4	<0.001
NEU ^b^, 10^9^ /L	7.38 ± 5.37	8.01 ± 3.11	5.24 ± 1.15	0.032
LYM ^b^, 10^9^ /L	1.39 ± 0.67	0.87 ± 0.43	1.65 ± 1.27	0.036
CRP (mg/L)	7.5 ± 2.7	8.7 ±3.3	7.2 ± 2.5	0.005
NLR <1 >1	1.23 (0.56)	1.65 (1.40, 2.09)	0.89 (0.75, 1.17)	<0.001
	24 (34.3%)	4 (28.6%)	20 (35.7%)	
	46 (65.7%)	10 (71.4%)	36 (64.3%)	

^a^Data expressed as mean ± SD for quantitative measures and also both number and percentage; ^b^Comparison using χ^2^ and quantitative variables using T test; Neutrophils (NEU), Lymphocyte (LYM), C-reactive protein (CRP).

**Figure 2:**
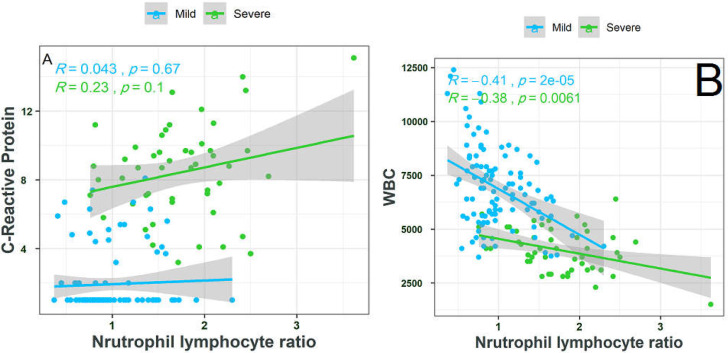
Association of NLR with CRP (A) and WBC levels (B) in severe and mild COVID-19 patients

**Figure 3:**
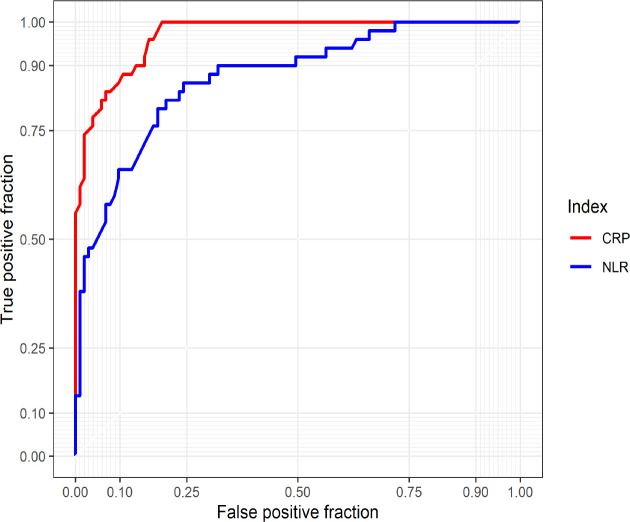
Area under the receiver operating characteristic curve of NLR and CRP in predicting cases with severe and mild COVID-19; CRP with AUC = 0.97 (95% CI: 0.95-0.99), and NLR with AUC = 0.87 (95% CI: 0.81-0.93).

Plasma CRP levels were higher in severe cases than in non-severe cases, and this difference was significant (*p*=0.005). The mean WBC and NLR of severe patients were significantly higher than those of non-severe patients (*p*<0.001). To identify the association of NLR with WBC and CRP levels in severe and non-severe patients, a correlation analysis was conducted (Figure 2). In severe patients, the results showed that NLR was positively correlated with CRP levels (R=0.23) and negatively correlated with WBC (R=-0.38).

Figure 3 shows the area under the ROC curve of the parameters in COVID-19 cases. CRP (AUC = 0.97, 95% CI: 0.95-0.99) and NLR (AUC = 0.87, 95% CI: 0.81-0.93) had very good accuracy in predicting the severity of COVID-19 disease.

## Discussion

COVID-19 has elicited a rapid spread of outbreak through human-to-human transmission, with an average incubation period of 5-6 days (14). Recent research has shown that 26% of patients received ICU care, and the mortality rate was less than 5% (15, 16).

The evidence suggests that early identification of critical illness and risk stratification management will reduce mortality and help alleviate the burden on insufficient medical resources. Recent studies have reported that low lymphocyte-to-C-reactive protein ratio and CRP could be predictive biomarkers for COVID-19 severity (17, 18). 

CRP is an acute-phase protein responsible for the clearance of pathogens through binding to pathogens and enhanced elimination by phagocytic cells. A positive correlation between CRP levels and lung lesions, kidney damage, and cardiac injury has been demonstrated; when the inflammation or tissue damage is resolved, CRP concentration falls (19, 20).

In the current study, a significantly higher CRP level (*p*=0.005) was observed in severe COVID-19 patients compared with the mild group, as previously reported by other studies (21- 23). As the first line of innate host defenses for clearance of viral infections, CRP might be linked to the overproduction of inflammatory cytokines in severe patients and may lead to dysfunction of various organ systems in COVID-19-infected patients (24, 25). 

NLR is widely used marker and defined by neutrophil count divided by lymphocyte count. The NLR index was found to be an indicator of prognosis in patients with pneumonia and tumors (26, 27). The findings have proven the hypothesis of the present study and suggest that elevated NLR is a functional biomarker that influences the progression of pneumonia in COVID-19 patients. These findings are supported by previous studies that have backed up the prognostic utility of NLR in COVID-19 patients (28, 29). Neutrophil (NEU) is a major component of the leukocyte population and can kill pathogens by releasing reactive oxygen species, producing effector molecules such as circulating vascular endothelial growth factor (VEGF), and inducing inflammatory factors as well as IL1, TNFα, and IFN-γ (30). Thus, because of the human immune response and cytokines produced by lymphocyte and endothelial cells, elevated NLR values may be seen following COVID-19 progression (31). In the present study, the ROC curve was used to analyze the specificity and sensitivity of different CRP and NLR values in severe and non-severe COVID-19 patients. The data showed that the AUC of NLR and CRP was 0.87 and 0.97, respectively. The AUC for NLR in this study was similar to a previous study by Yan Yufei et al., who reported it as 0.835, but it was more than that for CRP (0.775) (32). The AUC of the two parameters indicated that they could be used to predict the severity of COVID-19 disease.

The findings of this study indicate that the integration of NLR and CRP may lead to improved prediction and is thus recommended as a valuable early marker to assess prognosis and evaluate the severity of clinical symptoms in COVID-19 patients. Further research is needed to confirm the current findings and to compare the predictive ability of baseline NLR and the change in NLR under treatments. 
